# *RPGR*-Associated Dystrophies: Clinical, Genetic, and Histopathological Features

**DOI:** 10.3390/ijms21030835

**Published:** 2020-01-28

**Authors:** Xuan-Thanh-An Nguyen, Mays Talib, Mary J. van Schooneveld, Joost Brinks, Jacoline ten Brink, Ralph J. Florijn, Jan Wijnholds, Robert M. Verdijk, Arthur A. Bergen, Camiel J.F. Boon

**Affiliations:** 1 Department of Ophthalmology, Leiden University Medical Center, Albinusdreef 2, 2333 ZA Leiden, The Netherlands; x.nguyen@lumc.nl (X.-T.-A.N.); m.talib@lumc.nl (M.T.); j.brinks@lumc.nl (J.B.); j.wijnholds@lumc.nl (J.W.); 2 Department of Ophthalmology, Amsterdam UMC, Academic Medical Center, Meibergdreef 9, 1105 AZ Amsterdam, The Netherlands; m.j.vanschooneveld@amc.uva.nl; 3 Department of Clinical Genetics, Amsterdam UMC, Academic Medical Center, Meibergdreef 9, 1105 AZ Amsterdam, The Netherlands; j.b.tenbrink@amsterdamumc.nl (J.t.B.); r.j.florijn@amsterdamumc.nl (R.J.F.); aabergen@amc.uva.nl (A.A.B.); 4 Netherlands Institute for Neuroscience, an Institute of the Royal Netherlands Academy of Arts and Sciences (KNAW), Meibergdreef 47, 1105 BA Amsterdam, The Netherlands; 5 Department of Pathology, Leiden University Medical Center, Albinusdreef 2, 2333 ZA Leiden, The Netherlands; r.verdijk@erasmusmc.nl; 6 Department of Pathology, Section Ophthalmic Pathology, Erasmus MC University Medical Center, Doctor Molewaterplein 40, 3015 GD Rotterdam, The Netherlands

**Keywords:** retinitis pigmentosa, Retinitis Pigmentosa GTPase Regulator (RPGR), cone-rod dystrophy, histopathology, retinal dystrophies, natural history, genotype-phenotype

## Abstract

This study describes the clinical, genetic, and histopathological features in patients with *RPGR*-associated retinal dystrophies. Nine male patients from eight unrelated families underwent a comprehensive ophthalmic examination. Additionally, the histopathology of the right eye from a patient with an end-stage cone-rod-dystrophy (CRD)/sector retinitis pigmentosa (RP) phenotype was examined. All *RPGR* mutations causing a CRD phenotype were situated in exon ORF15. The mean best-corrected visual acuity (BCVA, decimals) was 0.58 (standard deviation (SD)): 0.34; range: 0.05–1.13); and the mean spherical refractive error was −4.1 D (SD: 2.11; range: −1.38 to −8.19). Hyperautofluorescent rings were observed in six patients. Full-field electroretinography responses were absent in all patients. The visual field defects ranged from peripheral constriction to central islands. The mean macular sensitivity on microperimetry was 11.6 dB (SD: 7.8; range: 1.6–24.4) and correlated significantly with BCVA (*r* = 0.907; *p* = 0.001). A histological examination of the donor eye showed disruption of retinal topology and stratification, with a more severe loss found in the peripheral regions. Reactive gliosis was seen in the inner layers of all regions. Our study demonstrates the highly variable phenotype found in *RPGR*-associated retinal dystrophies. Therapies should be applied at the earliest signs of photoreceptor degeneration, prior to the remodeling of the inner retina.

## 1. Introduction

Retinitis pigmentosa (RP) is the most common inherited retinal dystrophy, affecting approximately 1 in 3000 individuals [1]. Its predominant feature is the irreversible loss of rod photoreceptors, with secondary loss of cone photoreceptors. Patients therefore typically present with symptoms of nyctalopia and peripheral visual field constriction, prior to symptoms of central vision loss. X-linked RP (XLRP) accounts for 5%–15% of all RP cases and is recognized as one of the most severe forms of RP [2,3]. Mutations in the *RPGR* gene are responsible for 70%–90% of all XLRP cases [4,5,6]. Symptom onset in affected males starts in childhood years and is described to reach blindness within the 4th decade of life [2,7,8]. Despite the X-linked inheritance of *RPGR,* female carriers may also be affected by XLRP [9,10].

Other phenotypes caused by mutations in the *RPGR* gene include X-linked cone-rod dystrophies (CRD) and cone dystrophies (CD), in which degeneration predominantly affects cones, with or without the later involvement of rods [11]. Compared to XLRP, the age at onset is later in XL-CRD/CD, typically starting around the 4th decade of life, with initial symptoms being visual acuity loss, color vision defects, and variable photophobia [2,12]. 

The *RPGR* gene encodes the retinitis pigmentosa GTPase regulator (RPGR) protein and is able to express multiple isoforms through alternative splicing. The most common protein isoforms found in the retina are RPGR^ORF15^ and, to a lesser extent, the constitutive protein RPGR^1–19^ [13,14]. The RPGR^ORF15^ transcript, which is believed to play a key role in intraflagellar transport processes, contains exon 1–14 and exon ORF15, which is formed by alternatively spliced exon 15 and intron 15 [15]. Exon ORF15 consists of multiple acidic glutamate–glycine repeats, which promotes polymerase arrest and replication slippage, thus making it a mutational hotspot for XLRP [16]. CRD/CD-disease causing variants in *RPGR* are also located in the ORF15 region and are typically located at the 3′ end of exon ORF15 [17]. 

At present, no approved treatments for *RPGR*-associated retinal dystrophies are available. Recent advances have been made in animal models using subretinal *RPGR*-mediated gene therapy, which has shown an increase in structural and functional survival of photoreceptors [18,19]. These findings have paved the way for human *RPGR*-targeted gene therapy, and phase I/II/III clinical trials are currently ongoing (NCT03252847, NCT03116113 and NCT03316560) [20]. To facilitate a better understanding of *RPGR*-associated retinal dystrophies and in view of future therapies, this study provides an extensive prospective phenotypic evaluation of patients harboring mutations in the *RPGR* gene. In addition, we report the histological changes in the retina of a donor *RPGR* patient who had a clinical diagnosis of advanced CRD.

## 2. Results

### 2.1. Clinical Examination

In total, nine male patients from eight different families underwent a clinical evaluation, with a mean age of 30.2 years (standard deviation [SD]: 11.54; range: 17.8–48.9) at the most recent examination. Patients had a diagnosis of RP (*n* = 8) or CRD (*n* = 1). Eight different mutations were found, which were located in exon 1–14 (*n* = 3) or exon ORF15 (*n* = 5, including the CRD patient), and were generally frameshift mutations (*n* = 6), followed by nonsense (*n* = 1) and missense (*n* = 1) mutations (Table S1). An overview of the clinical findings is provided in Table 1.

**Figure 1:**
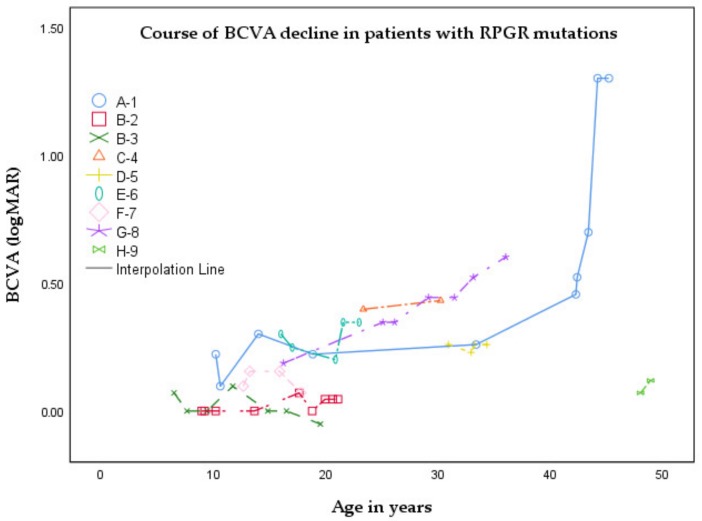
Graph demonstrating the change in mean best-corrected visual acuity (BCVA) in relation to the age in years of this cohort. Snellen BCVA data were transformed into logMAR values. Data from the same subject are shown using interpolation lines connecting the points. All patients had a retinitis pigmentosa phenotype, except for patient H-9, who exhibited a cone-rod dystrophy phenotype.

For RP patients, the mean age at onset was 6.1 years (SD: 2.8; range: 5.0–13.0), with initial symptoms being nyctalopia (*n* = 6; 75%) or visual field loss (*n* = 2; 25%). The mean best-corrected-visual-acuity (BCVA; decimals) was 0.57 (SD: 0.36; range: 0.05–1.13), and the mean spherical refractive error (SER) ranged from −1.88 D to −8.19 D (mean: −4.32 D; SD: 2.15). Besides the extensive prospective phenotypic evaluation, we were able to retrieve longitudinal BCVA data from the medical records of all patients with a mean follow-up of 11.5 years. In patient A-1, visual acuity remained relatively stable until the 4th decade of life, and declined afterwards. BCVA loss correlated significantly with increasing age (*r* = −0.857; *p* = 0.008) in patients with RP. In the CRD patient (H-9), the loss of visual acuity was the first symptom, which presented after the 4th decade of life. After initial presentation, BCVA loss (from 0.85 to 0.76) was already seen in a short time span of 0.9 years. The course of the BCVA decline of the entire cohort is presented in Figure 1.

**Figure 2:**
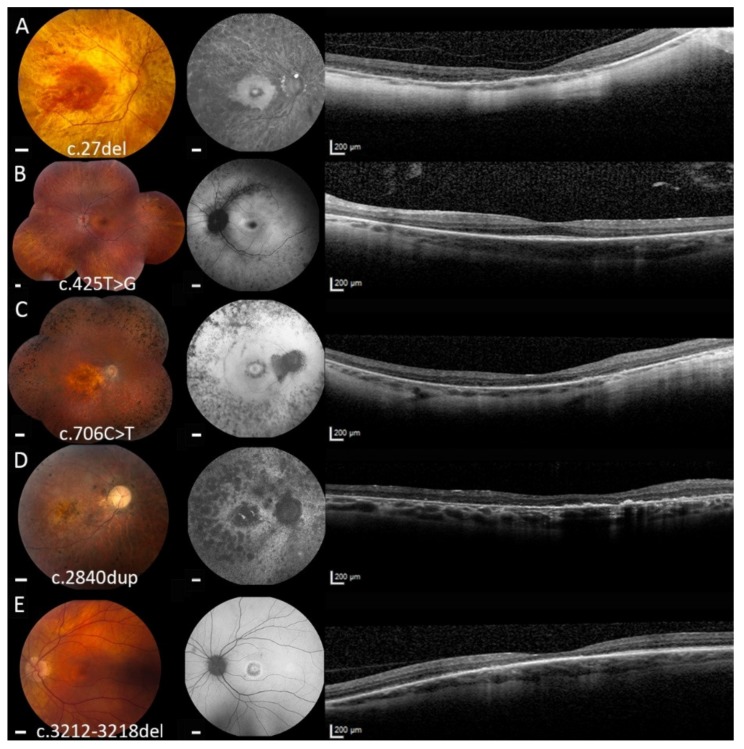
Color fundus photographs (scale bar = 1 mm) and corresponding fundus autofluorescence (FAF, scale bar = 1 mm) and spectral-domain optical coherence tomography (SD-OCT, scale bar = 200 µm) images of patients carrying mutations in the *RPGR* gene. (**A**) The right eye of patient A-1, with a best-corrected visual acuity (BCVA) of 0.05, showing a tilted optic disc, vascular attenuation, and bone-spicule hyperpigmentation in the midperiphery. Degenerative changes are seen across the entire retina, with sparing of the central macula. An atrophic perifoveal ring is present, resembling a bull’s eye maculopathy. Correspondingly, FAF imaging in this patient showed hypo-autofluorescent (hypo-AF) lesions throughout the posterior pole, with sparing of the central macula, and some optic disc drusen. On SD-OCT imaging, atrophy of outer retinal layers is observed, with some relative preservation of the ellipsoid zone (EZ) at the fovea. (**B**) The composite fundus photograph of the left eye of patient B-2 (BCVA of 0.9), showing optic nerve head drusen, normal vessels, and bone-spicule hyperpigmentation in the periphery. FAF imaging showed hypo-AF regions along the vascular arcades and far periphery. A macular hyperautofluorescent (hyper-AF) ring is observed, which matches the extent of EZ loss seen on SD-OCT. (**C**) The right eye of patient C-4 (BCVA of 0.36) carrying the c.706C > T (p.[Gln236*]) mutation. In addition to bone-spicule hyperpigmentation in the periphery, the fundus of this patient also displayed atrophic changes in the macula. A hyper-AF ring was seen on FAF, encircled by a hypo-AF ring, which corresponded with the loss of outer retinal bands on SD-OCT. (**D**) The right eye of patient G-8, with a BCVA of 0.25. The accumulation of hyperpigmented clumps in the macular region is observed, as well as outside the retinal vascular arcades. On FAF imaging, granular hypo-AF lesions are seen in the posterior pole, and a large central hypo-AF area is seen. SD-OCT imaging shows atrophy of retinal layers, with increased choroidal visibility. Remnants of the outer retinal bands are present in the (para)fovea, together with hyperreflective elevations at the level of the retinal pigment epithelium (RPE) that seem to correspond to the hyperpigmented deposits on fundus photography. (**E**) The left eye of patient H-9 (BCVA of 0.76), with a cone-rod dystrophy phenotype. A bull’s eye appearance is seen in the macula on fundus examination, without the presence of bone-spicule-like deposits. Similar findings are seen on FAF imaging, with hyper-AF spot in the fovea, where the outer retinal layers are relatively preserved on SD-OCT, surrounded by a hypo-AF ring of outer retinal and RPE atrophy, which itself is surrounded by a hyper-AF ring.

On fundoscopy, clinical hallmarks of RP, including optic disc pallor, vascular attenuation, and bone-spicule-like hyperpigmentation, were observed to various degrees (Figure 2). Optic nerve head drusen were seen in two patients. All patients showed macular abnormalities, ranging from moderate retinal pigment epithelium (RPE) changes to profound atrophy. Regions of RPE atrophy on fundus examination corresponded with the hypo-autofluorescent (hypo-AF) lesions seen on fundus autofluorescence (FAF) imaging. A macular hyperautofluorescent (hyper-AF) ring was seen in five out of eight RP patients. On spectral-domain optical coherence (SD-OCT) imaging, a common feature in RP patients was the loss of the outer retinal bands (external limiting membrane, ellipsoid zone and the inner/outer segments) in the retina peripheral of the central macula, with relative structural and functional sparing of the central macula. In the CRD patient, central macular atrophy was seen, with FAF showing a central hypo-AF area surrounded by a hyper-AF ring (Figure 2D). No bone-spicules were seen in this patient. SD-OCT imaging showed a loss of outer retinal bands focused at the (para)fovea. None of the patients had cystoid macular edema during examination.

**Figure 3:**
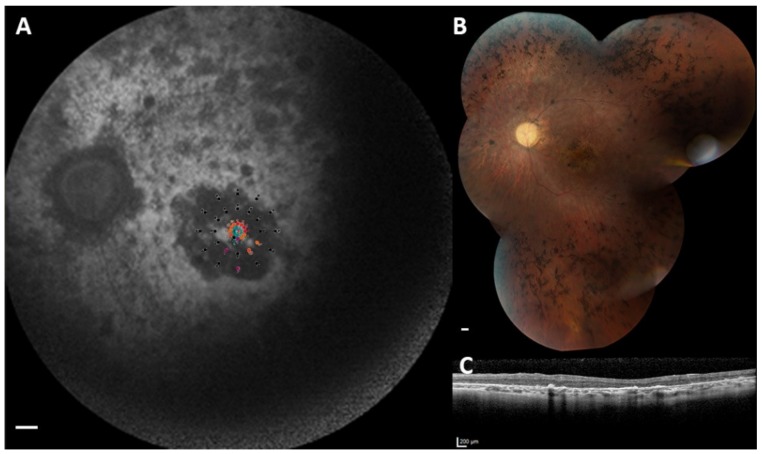
Multimodal imaging and microperimetry in the left eye of patient G-8 (best-corrected visual acuity of the left eye: 0.10). (**A**) Microperimetry revealed reduced, but measurable sensitivity (red and orange sensitivity points) at the central 2° of the fovea, with nearly a complete absence of sensitivity outside this region (black sensitivity points). Superimposed microperimetry data on the fundus autofluorescence (FAF) image demonstrated that loss of retinal sensitivity on microperimetry aligned with hypo-autofluorescent lesions on FAF. Scale bar = 1 mm. (**B**) The corresponding fundus image showed clinical hallmarks of retinitis pigmentosa and macular atrophy. Scale bar = 1 mm. (**C**) Spectral-domain optical coherence tomography (SD-OCT) showed remnants of the outer retina at the (para)fovea. Scale bar = 200 µm.

Full-field electroretinography (ffERG) responses were absent in all RP patients at the time of examination. Based on previous electroretinography (ERG) data (available for 5 RP patients), the mean age at which ERG amplitudes were found to be non-detectable was 11.6 years (SD: 6.7; range: 6.0–23.0). In the CRD patient, photopic responses were more severely reduced than scotopic responses, consistent with a CRD phenotype. We performed dark-adapted full-field stimulus thresholds (FST) testing in six patients using white and chromatic stimuli (Table S2). The mean general FST sensitivity for the white stimulus, averaged between eyes, was −31.1 dB (SD: 21.6; range: −61.0 to −8.9). The mean blue-red difference was 11.2 dB (SD: 10.9; range: −1.0 to 21.9), with most patients demonstrating a mixed rod-cone mediated response (*n* = 4, including the CRD patient) and two patients showing a cone-mediated response. The size of visual fields on kinetic perimetry (V4e stimuli), averaged between eyes, ranged between 42.0 and 997.7 mm^2^ (median: 153.8; interquartile range (IQR): 185.3), which correlated with the age of the RP patients (Spearman’s rho = −0.714; *p* = 0.047). Visual field abnormalities ranged from peripheral constriction to central islands in patients with RP, and a central scotoma in the CRD patient (Table 1). In addition, we successfully performed microperimetry testing in 16 eyes from nine patients (Table S2). Fixation stability was reported as stable (*n* = 12), relatively unstable (*n* = 2), or unstable (*n* = 2); the latter being the case in both eyes from patient A-1 (Snellen BCVA of 0.05 in both eyes). The stability of fixation, as measured using the 95% bivariate contour ellipse area (BCEA) values, correlated with BCVA (*r* = −0.926; *p* = 0.003). The mean retinal sensitivity was 11.6 dB (SD: 7.8; range: 1.6–24.4), which significantly correlated with BCVA (*r* = 0.907; *p* = 0.001) and not with the size of V4e retinal seeing fields (*r* = 0.553; *p* = 0.123). In RP patients with macular atrophy, some residual sensitivity at the central macula could still be observed (Figure 3).

### 2.2. Retinal Histology

**Figure 4:**
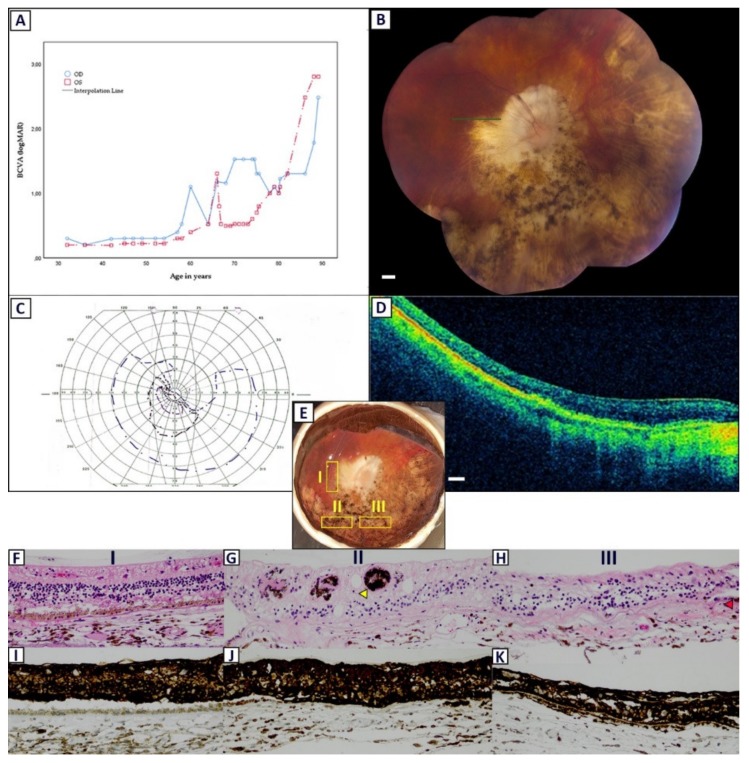
Clinical characteristics and postmortem histopathological examination in patient I-10 at the age of 94, who carried the p.Glu1031Glyfs*58 (c.3092del) mutation in *RPGR*. (**A**) A line graph detailing the best-corrected visual acuity (logMAR) course regression in this patient. A cataract extraction was performed at the age of 66 (left eye; OS) and 74 (right eye; OD). For hand movement vision, light perception vision, and no light perception, logMAR values of 2.7, 2.8, and 2.9 were used, respectively. (**B**) A fundus examination of the right eye at age 89 showed a pale optic disc with extensive peripapillary and macular atrophy. Retinal vessels were attenuated or obliterated. Bone-spicule-like pigmentation is mainly seen in the inferior-nasal quadrant. The green line illustrates the spectral-domain optical coherence tomography (SD-OCT) section line. Scale bar = 1 mm. (**C**) Goldmann kinetic perimetry at the age of 67 showed a superior hemifield defect. (**D**) SD-OCT imaging revealed profound degeneration of all retinal layers. Scale bar = 200 µm. (**E**) A macroscopic examination of the right eye. A schematic drawing indicating the approximate macular (I) and peripheral (II and III) regions sectioned and processed for further examination. (**F**) A section of the macular region (I) showing the loss of photoreceptor segments and the closing of the subretinal space. The retinal pigment epithelium (RPE) layer is atrophic, but still visible in this section (hematoxylin-eosin (H&E), 200×). (**G**) A complete absence of photoreceptor outer segments and RPE cells is observed in this peripheral section (II). The inner layers are highly atrophic and disorganized. The migration of RPE cells into retinal vessels is shown (yellow arrowhead; H&E, 200×). (**H**) A clear disruption of the normal topology and stratification is also seen in this section (III), although there are still some RPE cells remaining (red arrowhead (H&E, 200×)). (**I–K**) A positive immunoreactivity for glial fibrillary acid protein (GFAP) is seen across all retinal layers in both macular and peripheral regions (GFAP, 200×).

Medical records of the donor patient (I-10), carrying the p.Glu1031Glyfs*58 (c.3092del) *RPGR* mutation, were obtained. First, available records of this patient were found at the age of 32, with BCVA loss as initial symptom (BCVA OD: 0.5; BCVA OS: 0.63). ERG examination revealed non-detectable photopic responses and reduced scotopic amplitudes, suggesting a CRD phenotype. Goldmann kinetic perimetry (V4e stimulus) showed a superior hemifield defect (Figure 4C). BCVA remained relatively stable, until the patient revisited the clinic at the age of 58 (BCVA OD: 0.3; BCVA OS: 0.5). Cataract surgery due to a posterior subcapsular cataract in the left eye resulted in the improvement of BCVA in the left eye, but no BCVA improvement was seen in the right eye after uncomplicated cataract surgery. The course of BCVA regression/improvement of this patient is shown in Figure 4A. The last ophthalmic examination was available at the age of 89. At the last examination, only hand movements and light perception were observed in the right and left eye, respectively. On fundus examination, peripapillary atrophy extending to the macular region was seen. Bone-spicules were present mostly in the inferior and nasal quadrants, suggesting a mixed CRD /sector RP phenotype (Figure 4B). The right eye of patient I-10 was retrieved postmortem at the age of 94, and was prepared for histologic examination.

Sections of the macular and peripheral regions were processed for microscopic examination. Gross examination of the right eye showed bone-spicule-like pigmentation predominantly in the inferonasal quadrant (Figure 4E). Due to profound atrophy in the macular region, it was difficult to accurately distinguish and pinpoint the fovea. In all sections, degenerative changes and disorganization of the retinal laminae were evident. In the macular sections, the loss of photoreceptor outer segments was observed, which was accompanied by a reduction in the number of photoreceptor cells and the closing of the subretinal space. While the retinal pigment RPE layer was atrophic, it was not entirely lost in the macular region. 

Peripheral regions showed more extensive retinal remodeling than the macular region, with severe disruption of the normal topology and stratification. The complete loss of the photoreceptor layer and a major reduction in the RPE layer was observed. Some remaining patches of the RPE layer were still visible (Figure 4H, red arrowhead). In sections devoid of a pigmented RPE layer, RPE cells migrated and accumulated around the retinal vessels in the inner retina (Figure 4G, yellow arrowhead). Immunohistochemical staining showed positivity for glial fibrillary acid protein (GFAP) across the entire retina, in both macular and peripheral sections (Figure 4I–K), signifying reactive gliosis.

## 3. Discussion

In this study, we describe the clinical and genetic characteristics of *RPGR* patients at different stages of disease based on extensive prospective structural and functional phenotyping. The patients in this cohort demonstrated typical features of RP or CRD. An early onset of symptoms was present in patients with RP, with initial symptoms being night blindness or peripheral visual field loss. As such, we found moderate to severe concentric visual field defects in all patients. In contrast, the first symptoms occurred at the age of 48 in the CRD patient. After the onset of the disease, BCVA gradually declined in the majority of patients. The fastest BCVA decline was seen in patient A-1, who demonstrated a rapid decline of BCVA after the 4th decade of life. A fundus examination revealed profound atrophy in the macula of this patient. While macular involvement occurs early in CRD, it typically is not seen in RP patients until end-stage disease [12,21]. Retinal imaging in this study showed RPE atrophy on FAF in various patterns and the loss of outer retinal bands on SD-OCT, which were all consistent with either an RP or CRD phenotype. A hyper-AF ring was found in six out of nine patients, including the CRD patient. Previous studies have shown that the hyper-AF ring correlates with the presence of the EZ band and demarcates the transition between healthy and affected retina [2,22,23]. The constriction of this hyper-AF ring in RP indicates disease progression, whereas, conversely, the expansion of the hyper-AF ring in CRD patients suggests disease progression [2,24]. 

Defining disease severity is essential for gene therapy trials, as preserved rods and cones are required for successful subretinal delivery [25]. For the assessment of rod and cone function, several functional and psychophysical measurement tools exist, and these methods were explored in this study. Microperimetry measures the retinal sensitivity of the macular region and is able to detect changes within a short time span [26]. In our cohort, we were able to detect retinal sensitivity loss in all patients, which correlated with the loss of visual acuity. Patients with macular atrophy still retained some residual function at the fovea, although severely reduced, which may be targeted for treatment. The benefit of microperimetry in gene therapy trials is the ability to measure the individual retinal points exposed to treatment and to correlate these with fundus locations. However, possible challenges with microperimetry may arise when patients with end-stage disease are included in clinical trials. Our study and others have found that fixation stability is correlated with visual acuity [27,28]. Fixation instability causes greater variation in measurements, which may impact the repeatability and reliability of testing [27,28]. Therefore, inherent test-retest variation should be taken into account when assessing retinal function with microperimetry. FST is a psychophysical test that measures the sensitivity of the entire retina, even in those without adequate fixation capabilities, and in patients who do not have measurable rod and cone ffERG responses [29,30]. Using FST, we found mixed rod–cone-mediated responses in four patients (including the CRD patient), and cone-mediated responses in two RP patients. Patients with cone-mediated responses in our cohort showed macular atrophy and widespread RPE loss, suggesting that cone-mediated responses are indicators for more advanced disease stages of RP [29]. Other studies have shown a strong correlation between FST and residual ffERG amplitudes, suggesting that FST can be a potential replacement for ERG [31,32]. In patients with *RPGR*-associated RP, ERG responses are often severely reduced or absent from an early age, as was the case in our cohort, and may not be an optimal parameter for future trials. FST could potentially replace ERG as a psychophysical outcome for future *RPGR*-related studies and is especially useful in patients with severe disease and fixation instability. However, as FST measures the entire retina, it is unable to localize the individual retinal areas mediating the responses. Therefore, caution must be exercised when interpreting FST results in clinical trials, as they may not co-localize with the area of treatment. 

Some possible genotype–phenotype correlations could be identified within this cohort. Nine different variants in *RPGR* were detected, including the mutation found in the donor patient (I-10). Frameshift mutations were the most common mutation type and were mainly located in exon ORF15. It is worth noting that the mutations causing CRD phenotypes, p.(Glu1031Glyfs*58, the eye of patient I-10 in whom histopathological studies were performed) and p.(Glu1071Alafs*16, patient H-9), were found at the 3′ of ORF15. Our data support previous studies that suggested that mutations at the 3′ terminal of ORF15 cause predominant CRD phenotypes [4,17]. It is believed that frameshift mutations in the exons that encode the N-terminal RCC1-like domain of RPGR are more prone to nonsense-mediated decay (NMD), leading to lower levels of the transcript and may therefore be more likely to cause severe RP phenotypes [4]. Furthermore, the N-terminal RCC1-like domain of RPGR is shared between RPGR^ORF15^ and RPGR^1–19^ isoforms. Since RPGR^1–19^ is also expressed in primary cilia, it is believed that mutations in the N-terminal RCC1-like domain may lead to retinal degeneration with ciliary abnormalities, as shown in previous studies [33,34,35]. This phenotype was not observed in our cohort, although more thorough testing is required to confirm the absence of ciliary defects. Mutations in ORF15 are located at the terminal exon and are less likely to result in NMD, resulting in milder RP (towards the 5′ end) and CRD (towards the 3′ end) phenotypes [4,17]. This is not always the case, as mutations located close to the downstream of ORF15 have also been reported to result in RP phenotypes [2,36]. In some cases, ORF15 mutations even resulted in both RP and CRD phenotypes, highlighting the potential influence of genetic and/or environmental modifiers on the phenotype [16].

We also evaluated the retinal histopathology of an affected 94-year old patient with an end-stage CRD/sector RP phenotype. There have only been a few histopathologic studies on eyes from patients with known *RPGR* mutations [37,38]. Similar to CRD patient H-9, initial symptoms presented at a much later time, at the age of 32, than the RP patients in this cohort. The longitudinal BCVA follow-up of 57 years in this patient showed that visual acuity gradually deteriorated. Strikingly, fundus examination in this patient showed extensive peripapillary atrophy and bone-spicule hyperpigmentation, mainly in the nasal and inferior quadrants, corresponding with a superior hemifield defects on kinetic perimetry. Predisposition for superior visual field loss has been described in patients with sector RP caused by mutations in the *RHO* gene, and rarely in patients with *RPGR* mutations [25,39,40]. Sectoral changes are believed to reflect a milder phenotype and can develop into a widespread disease when followed over decades [39]. On histologic examination, we found a severely atrophic and disorganized retina in all sections. There was a widespread loss of photoreceptors, more so in the peripheral retina than in the macula, which may explain the remaining visual acuity of only hand movements in this patient’s right eye. Immunohistochemical analysis showed an intense staining for GFAP across the entire inner retina in the peripheral region, indicating the process of reactive gliosis by Müller cells [41]. Inner remodeling is detrimental for the application of bionic (retinal chip) and biological (gene, optogenetic, or stem cell) rescue strategies. These therapies rely on remaining target neurons and may have limited success when glial seal formation has taken place [42,43]. Therapies should therefore preferably be applied at the earlier signs of degeneration, prior to inner retinal remodeling. Interestingly, GFAP staining was also seen in macular sections, which revealed less degeneration compared to peripheral sections and showed no bone-spicule-like deposits on fundus examination. This suggests that the remodeling of the inner retina may already take place before clinical signs are observable on fundoscopy and retinal imaging [43]. No clinical imaging techniques exist that can accurately track the earliest stages of remodeling, which implicates the optimal timing for therapy [43]. The exact impact of retinal remodeling and gliosis on the long-term efficacy of therapeutic strategies is still unclear and needs to be further elucidated. 

In conclusion, we present a comprehensive clinical, genetic, and histopathologic overview of patients with *RPGR*-associated retinal dystrophies. The clinical presentation of *RPGR*-related retinal dystrophies is highly variable, but genotype–phenotype correlations can be discerned. We found that microperimetry and FST can be useful functional parameters for evaluation in future trials. In end-stage disease, remodeling takes place also in the inner retina, which may precede clinically observable signs, complicating future therapeutic strategies. Therefore, treatments should preferably be applied in an early disease stage.

## 4. Materials and Methods 

### 4.1. Clinical Examination

Male participants were ascertained from the RD5000 registry, which is a national registry for inherited retinal dystrophies [44]. Comprehensive ophthalmologic assessment was performed at the Leiden University Medical Center (LUMC), including the measurement of BCVA using the Early Treatment of Diabetic Retinopathy Study (ETDRS) chart, slit-lamp examination, color vision testing (Hardy-Rand-Rittler (HRR)), and fundus photography (Topcon TRC-50DX, Topcon Medical Systems, Inc. Oakland, NJ, USA). SD-OCT and FAF imaging were performed using the Spectralis HRA+OCT system (Heidelberg Engineering, Heidelberg, Germany). 

ffERG was performed according to International Society for Clinical Electrophysiology of Vision (ISCEV) standards. ffERG was not conducted in patients that were previously reported to have non-recordable responses (*n* = 5). FST testing was performed in 6 patients using the Espion Colordome^TM^ LED full-field stimulator (Diagnosys LCC, Lowell, MA, USA). Both eyes were tested, first using the white stimulus, followed by red and blue stimuli. Thresholds were tested three times for each color and were averaged to determine the final thresholds. The difference in thresholds between blue and red stimuli determined whether responses were either: rod-mediated (difference of > 22 dB), cone-mediated (difference of < 3 dB), or mixed (difference between 3 and 22 dB) [29]. Goldmann kinetic perimetry was performed using V4e stimuli, and visual fields were digitized using a method previously used by Dagnelie [45]. In addition, microperimetry (MAIA, Centervue, Padova, Italy) was performed in all patients, under mesopic conditions, by performing the “4–2 fixed protocol” to minimize a learning effect, followed by the “4–2 strategy” protocol for formal testing. 

The present study was approved by the Medical Ethics Committee of the Erasmus Medical Center (MEC-2010-359, approval date: 10/04/2015), as well by the Local Review Board of the LUMC (P11.100, approval date: 09/11/2015). Informed consent was retrieved for all participants, and the study adhered to the tenets of the Declarations of Helsinki. When available, clinical data from medical records were retrieved for longitudinal evaluation.

### 4.2. Retinal Histology

The right eye was obtained 15 h postmortem from a 94-year old male patient (I-10) with a confirmed mutation in the *RPGR* gene. Prior written informed consent for organ and tissue use in research was given by the donor. Relevant tissues were fixed in 4% paraformaldehyde for 24 h and embedded in paraffin. The embedded blocks were cut at 4 µm sections and stained with hematoxylin-eosin. For immunohistochemical staining, following deparaffinization, rehydration, and heat-induced epitope retrieval, the tissue sections were incubated with primary glial fibrillary acid protein (GFAP; monoclonal mouse antibody, clone 6f2; DakoCytomation, Glostrup, Denmark; dilution 1:400) for 30 min and were counterstained with hematoxylin [46]. The slides were externally validated using appropriate control tissues and were reviewed by an ophthalmic pathologist (R.M.V).

### 4.3. Genetic Analysis

Genomic DNA was extracted from peripheral blood samples according to standard protocols. Genetic analysis was performed at the Department of Clinical Genetics of the Amsterdam University Medical Centers (Amsterdam UMC, The Netherlands), the Netherlands, and methods used have been published previously [2,10,47]. The primers used in this study can be accessed upon reasonable request. 

### 4.4. Statistical Analysis

Data were analyzed using Statistical Package for the Social Sciences version 25.0 (IBM Corp, Armonk, NY, USA). Findings with a *p*-value of <0.05 were considered statistically significant. Normally and non-normally distributed data were displayed as means with SD and medians with IQR, respectively. Depending on the distribution, either a Pearson’s or Spearman’s test was performed for correlation testing.

## Figures and Tables

**Table 1:** Clinical characteristics of patients with *RPGR*-associated retinal dystrophies at the last examination.

Family-ID	Age	Age at Onset	Initial Symptom	DD	BCVA		SER		Lens Status		Fundus Features				Goldmann Perimetry (V4e)		
					OD	OS	OD	OS	OD	OS	Optic Pallor	Attenuated Vessels	Bone-Spicules	Other Relevant Findings	Visual Field Patterns	Retinal Seeing Retinal Areas (mm^2^) OD|OS	
A-1	45	5	VF loss	RP	0.05	0.05	−1.0	−2.75	Mild PSC	Mild PSC	Yes	Yes	Yes	Bull’s eye appearance of macula	Central island with peripheral remnant	95.3	120.4
B-2 *	20	5	Night blindness	RP	0.80	1.00	−5.00	−5.13	Clear	Clear	Yes	No	Yes	Optic disc drusen	Peripheral constriction	532.7	481.0
B-3 *	20	6	Night blindness	RP	1.25	1.00	−1.88	−0.88	Clear	Clear	Yes	No	Yes	Optic disc drusen	Central island with peripheral remnant	148.8	158.7
C-4	30	5	Night blindness	RP	0.36	0.38	−5.63	−4.88	Clear	Clear	Yes	Yes	Yes	Macular atrophy	Central islands	84.6	73.8
D-5	33	13	VF loss	RP	0.66	0.52	−5.75	−4.38	Clear	Clear	Yes	Yes	Yes	Patches of preserved RPE	Central island with peripheral remnant	279.5	247.6
E-6	22	5	Night blindness	RP	0.50	0.40	−4.13	−4.50	Clear	Clear	Yes	Yes	Yes	Epiretinal membrane	Central island with peripheral remnant	115.2	101.83
F-7	18	5	Night blindness	RP	0.70	1.00	−8.88	−7.50	Clear	Clear	Yes	Yes	Yes	RPE alterations	Midperipheral scotoma	632.2	669.5
G-8	36	5	Night blindness	RP	0.10	0.10	−3.63	−3.25	Clear	Clear	Yes	Yes	Yes	Macular atrophy	Central island	24.8	39.2
H-9	49	48	VA loss	CRD	0.80	0.72	−3.25	−1.50	Clear	Clear	Yes	No	No	Bull’s eye appearance of macula	Central scotoma	1213.32	782.04

DD = diagnosis; BCVA = best-corrected visual acuity; OD = right eye; OS = left eye; SER = spherical refractive error; VF = visual field; RP = retinitis pigmentosa; PSC = posterior subcapsular cataract; BS = bone-spicule-like pigmentation; NR = non-recordable responses; RPE = retinal pigment epithelium; VA = visual acuity; CRD = cone-rod dystrophy; HM = hand movements; LP = light perception vision. BCVA is shown in Snellen decimals. Visual fields were digitized into retinal seeing areas. * Patient B-2 and B-3 are 2nd degree relatives.

## Supplementary Materials

Supplementary materials can be found at https://www.mdpi.com/1422-0067/21/3/835/s1.

## Abbreviations

BCVA	Best-corrected visual acuity
CD	Cone dystrophy
CRD	Cone-rod dystrophy
FAF	Fundus autofluorescence
ffERG	Full-field electroretinography
FST	Full-stimulus threshold
GFAP	Glial fibrillary acid protein
Hyper-AF	Hyperautofluorescent
Hypo-AF	Hypo-autofluorescent
IQR	Interquartile range
RP	Retinitis pigmentosa
RPE	Retinal pigment epithelium
RPGR	Retinitis Pigmentosa GTPase Regulator
SD	Standard deviation
SD-OCT	Spectral-Domain Optical Coherence Tomography
XLRP	X-linked retinitis pigmentosa

## References

[1] Hartong D.T., Berson E.L., Dryja T.P. (2006). Retinitis pigmentosa. Lancet.

[2] Talib M., van Schooneveld M.J., Thiadens A.A., Fiocco M., Wijnholds J., Florijn R.J., Schalij-Delfos N.E., van Genderen M.M., Putter H., Cremers F.P.M. (2019). Clinical and Genetic characteristics of Male patients with RPGR-associated Retinal Dystrophies: A long-Term Follow-up Study. RETINA.

[3] Tee J.J.L., Yang Y., Kalitzeos A., Webster A., Bainbridge J., Weleber R.G., Michaelides M. (2018). Characterization of Visual Function, Interocular Variability and Progression Using Static Perimetry–Derived Metrics in RPGR-Associated Retinopathy. Investig. Ophthalmol. Vis. Sci..

[4] Sharon D., Sandberg M.A., Rabe V.W., Stillberger M., Dryja T.P., Berson E.L. (2003). RP2 and RPGR Mutations and Clinical Correlations in Patients with X-Linked Retinitis Pigmentosa. Am. J. Hum. Genet..

[5] Bader I., Brandau O., Achatz H., Apfelstedt-Sylla E., Hergersberg M., Lorenz B., Wissinger B., Wittwer B.r., Rudolph G.n., Meindl A. (2003). X-linked Retinitis Pigmentosa: RPGR Mutations in Most Families with Definite X Linkage and Clustering of Mutations in a Short Sequence Stretch of Exon ORF15. Investig. Ophthalmol. Vis. Sci..

[6] Pelletier V., Jambou M., Delphin N., Zinovieva E., Stum M., Gigarel N., Dollfus H., Hamel C., Toutain A., Dufier J.-L. (2007). Comprehensive survey of mutations in RP2 and RPGR in patients affected with distinct retinal dystrophies: Genotype–phenotype correlations and impact on genetic counseling. Hum. Mutat..

[7] Bird A.C. (1975). X-linked retinitis pigmentosa. Br. J. Ophthalmol..

[8] Sandberg M.A., Rosner B., Weigel-DiFranco C., Dryja T.P., Berson E.L. (2007). Disease course of patients with X-linked retinitis pigmentosa due to RPGR gene mutations. Investig. Ophthalmol. Vis. Sci..

[9] Nanda A., Salvetti A.P., Clouston P., Downes S.M., MacLaren R.E. (2018). Exploring the Variable Phenotypes of RPGR Carrier Females in Assessing their Potential for Retinal Gene Therapy. Genes.

[10] Talib M., van Schooneveld M.J., Van Cauwenbergh C., Wijnholds J., ten Brink J.B., Florijn R.J., Schalij-Delfos N.E., Dagnelie G., van Genderen M.M., De Baere E. (2018). The Spectrum of Structural and Functional Abnormalities in Female Carriers of Pathogenic Variants in the RPGR Gene. Investig. Ophthalmol. Vis. Sci..

[11] Ebenezer N.D., Michaelides M., Jenkins S.A., Audo I., Webster A.R., Cheetham M.E., Stockman A., Maher E.R., Ainsworth J.R., Yates J.R. (2005). Identification of Novel RPGR ORF15 Mutations in X-linked Progressive Cone-Rod Dystrophy (XLCORD) Families. Investig. Ophthalmol. Vis. Sci..

[12] Thiadens A.A.H.J., Soerjoesing G.G., Florijn R.J., Tjiam A.G., den Hollander A.I., van den Born L.I., Riemslag F.C., Bergen A.A.B., Klaver C.C.W. (2011). Clinical course of cone dystrophy caused by mutations in the RPGR gene. Graefes Arch. Clin. Exp. Ophthalmol..

[13] Megaw R.D., Soares D.C., Wright A.F. (2015). RPGR: Its role in photoreceptor physiology, human disease, and future therapies. Exp. Eye Res..

[14] Kirschner R., Rosenberg T., Schultz-Heienbrok R., Lenzner S., Feil S., Roepman R., Cremers F.P.M., Ropers H.-H., Berger W. (1999). RPGR Transcription Studies in Mouse and Human Tissues Reveal a Retina-Specific Isoform That Is Disrupted in a Patient With X-Linked Retinitis Pigmentosa. Hum. Mol. Genet..

[15] Hosch J., Lorenz B., Stieger K. (2011). RPGR: Role in the photoreceptor cilium, human retinal disease, and gene therapy. Ophthalmic Genet..

[16] Yang L., Yin X., Feng L., You D., Wu L., Chen N., Li A., Li G., Ma Z. (2014). Novel Mutations of RPGR in Chinese Retinitis Pigmentosa Patients and the Genotype-Phenotype Correlation. PLoS ONE.

[17] Demirci F.Y.K., Rigatti B.W., Wen G., Radak A.L., Mah T.S., Baic C.L., Traboulsi E.I., Alitalo T., Ramser J., Gorin M.B. (2002). X-Linked Cone-Rod Dystrophy (Locus COD1): Identification of Mutations in RPGR Exon ORF15. Am. J. Hum. Genet..

[18] Beltran W.A., Cideciyan A.V., Lewin A.S., Iwabe S., Khanna H., Sumaroka A., Chiodo V.A., Fajardo D.S., Román A.J., Deng W.-T. (2012). Gene therapy rescues photoreceptor blindness in dogs and paves the way for treating human X-linked retinitis pigmentosa. Proc. Natl. Acad. Sci. USA.

[19] Fischer M.D., McClements M.E., Martinez-Fernandez de la Camara C., Bellingrath J.-S., Dauletbekov D., Ramsden S.C., Hickey D.G., Barnard A.R., MacLaren R.E. (2017). Codon-Optimized RPGR Improves Stability and Efficacy of AAV8 Gene Therapy in Two Mouse Models of X-Linked Retinitis Pigmentosa. Mol. Ther..

[20] Cehajic Kapetanovic J., McClements M.E., Martinez-Fernandez de la Camara C., MacLaren R.E. (2019). Molecular Strategies for RPGR Gene Therapy. Genes.

[21] Van Huet R.A.C., Estrada-Cuzcano A., Banin E., Rotenstreich Y., Hipp S., Kohl S., Hoyng C.B., den Hollander A.I., Collin R.W.J., Klevering B.J. (2013). Clinical Characteristics of Rod and Cone Photoreceptor Dystrophies in Patients With Mutations in the C8orf37 Gene. Investig. Ophthalmol. Vis. Sci..

[22] Tee J.J.L., Yang Y., Kalitzeos A., Webster A., Bainbridge J., Michaelides M. (2019). Natural History Study of Retinal Structure, Progression, and Symmetry Using Ellipzoid Zone Metrics in RPGR-Associated Retinopathy. Am. J. Ophthalmol..

[23] Lima L.H., Burke T., Greenstein V.C., Chou C.L., Cella W., Yannuzzi L.A., Tsang S.H. (2012). Progressive Constriction of the Hyperautofluorescent Ring in Retinitis Pigmentosa. Am. J. Ophthalmol..

[24] Barnes C.S., Schuchard R.A., Birch D.G., Dagnelie G., Wood L., Koenekoop R.K., Bittner A.K. (2019). Reliability of Semiautomated Kinetic Perimetry (SKP) and Goldmann Kinetic Perimetry in Children and Adults With Retinal Dystrophies. Transl. Vis. Sci. Technol..

[25] Charng J., Cideciyan A.V., Jacobson S.G., Sumaroka A., Schwartz S.B., Swider M., Roman A.J., Sheplock R., Anand M., Peden M.C. (2016). Variegated yet non-random rod and cone photoreceptor disease patterns in RPGR-ORF15-associated retinal degeneration. Hum Mol Genet.

[26] Bagdonaite-Bejarano L., Hansen R.M., Fulton A.B. (2019). Microperimetry in Three Inherited Retinal Disorders. Semin. Ophthalmol..

[27] Jolly J.K., Xue K., Edwards T.L., Groppe M., MacLaren R.E. (2017). Characterizing the Natural History of Visual Function in Choroideremia Using Microperimetry and Multimodal Retinal Imaging. Investig. Ophthalmol. Vis. Sci..

[28] Dimopoulos I.S., Tseng C., MacDonald I.M. (2016). Microperimetry as an Outcome Measure in Choroideremia Trials: Reproducibility and Beyond. Investig. Ophthalmol. Vis. Sci..

[29] Jacobson S.G., Aleman T.S., Cideciyan A.V., Roman A.J., Sumaroka A., Windsor E.A.M., Schwartz S.B., Heon E., Stone E.M. (2009). Defining the Residual Vision in Leber Congenital Amaurosis Caused by RPE65 Mutations. Investig. Ophthalmol. Vis. Sci..

[30] Russell S., Bennett J., Wellman J.A., Chung D.C., Yu Z.-F., Tillman A., Wittes J., Pappas J., Elci O., McCague S. (2017). Efficacy and safety of voretigene neparvovec (AAV2-hRPE65v2) in patients with RPE65-mediated inherited retinal dystrophy: A randomised, controlled, open-label, phase 3 trial. Lancet.

[31] Roman A.J., Cideciyan A.V., Aleman T.S., Jacobson S.G. (2007). Full-field stimulus testing (FST) to quantify visual perception in severely blind candidates for treatment trials. Physiol. Meas..

[32] Messias K., Jägle H., Saran R., Ruppert A.D.P., Siqueira R., Jorge R., Messias A. (2013). Psychophysically determined full-field stimulus thresholds (FST) in retinitis pigmentosa: Relationships with electroretinography and visual field outcomes. Doc. Ophthalmol..

[33] Zito I., Downes S.M., Patel R.J., Cheetham M.E., Ebenezer N.D., Jenkins S.A., Bhattacharya S.S., Webster A.R., Holder G.E., Bird A.C. (2003). RPGR mutation associated with retinitis pigmentosa, impaired hearing, and sinorespiratory infections. J. Med Genet..

[34] Iannaccone A., Breuer D.K., Wang X.F., Kuo S.F., Normando E.M., Filippova E., Baldi A., Hiriyanna S., MacDonald C.B., Baldi F. (2003). Clinical and immunohistochemical evidence for an X linked retinitis pigmentosa syndrome with recurrent infections and hearing loss in association with an RPGR mutation. J. Med Genet..

[35] Van Dorp D.B., Wright A.F., Carothers A.D., Bleeker-Wagemakers E.M. (1992). A family with RP3 type of X-linked retinitis pigmentosa: An association with ciliary abnormalities. Hum. Genet..

[36] Vervoort R., Lennon A., Bird A.C., Tulloch B., Axton R., Miano M.G., Meindl A., Meitinger T., Ciccodicola A., Wright A.F. (2000). Mutational hot spot within a new RPGR exon in X-linked retinitis pigmentosa. Nat. Genet..

[37] Aguirre G.D., Yashar B.M., John S.K., Smith J.E., Breuer D.K., Hiriyanna S., Swaroop A., Milam A.H. (2002). Retinal Histopathology of an XLRP Carrier with a Mutation in the RPGR Exon ORF15. Exp. Eye Res..

[38] Huang W.C., Wright A.F., Roman A.J., Cideciyan A.V., Manson F.D., Gewaily D.Y., Schwartz S.B., Sadigh S., Limberis M.P., Bell P. (2012). RPGR-associated retinal degeneration in human X-linked RP and a murine model. Investig. Ophthalmol. Vis. Sci..

[39] Ramon E., Cordomí A., Aguilà M., Srinivasan S., Dong X., Moore A.T., Webster A.R., Cheetham M.E., Garriga P. (2014). Differential light-induced responses in sectorial inherited retinal degeneration. J Biol Chem.

[40] Kranich H., Bartkowski S., Denton M.J., Krey S., Dickinson P., Duvigneau C., Gal A. (1993). Autosomal dominant ‘sector’ retinitis pigmentosa due to a point mutation predicting an Asn-15-Ser substitution of rhodopsin. Hum. Mol. Genet..

[41] Henriksen B.S., Marc R.E., Bernstein P.S. (2014). Optogenetics for retinal disorders. J Ophthalmic Vis Res.

[42] Marc R.E., Jones B.W., Watt C.B., Strettoi E. (2003). Neural remodeling in retinal degeneration. Prog. Retin. Eye Res..

[43] Jones B.W., Pfeiffer R.L., Ferrell W.D., Watt C.B., Marmor M., Marc R.E. (2016). Retinal remodeling in human retinitis pigmentosa. Exp. Eye Res..

[44] Van Huet R.A.C., Oomen C.J., Plomp A.S., van Genderen M.M., Klevering B.J., Schlingemann R.O., Klaver C.C.W., van den Born L.I., Cremers F.P.M. (2014). The RD5000 Database: Facilitating clinical, genetic, and therapeutic studies on inherited retinal diseases. Investig. Ophthalmol. Vis. Sci..

[45] Dagnelie G. (1990). Technical note. Conversion of planimetric visual field data into solid angles and retinal areas. Clin. Vis. Sci..

[46] Bais B., Kubat B., Motazedi E., Verdijk R.M. (2015). Amyloid Precursor Protein and Ubiquitin Immunohistochemistry Aid in the Evaluation of Infant Autopsy Eyes With Abusive Head Trauma. Am. J. Ophthalmol..

[47] Roepman R., Bernoud-Hubac N., Schick D.E., Maugeri A., Berger W., Ropers H.-H., Cremers F.P.M., Ferreira P.A. (2000). The retinitis pigmentosa GTPase regulator (RPGR) interacts with novel transport-like proteins in the outer segments of rod photoreceptors. Hum. Mol. Genet..

