# Supplementary material for: RPGR-Associated Dystrophies: Clinical, Genetic, and Histopathological Features
Source: Int J Mol Sci. 2020 Jan 28;21(3):835. doi: 10.3390/ijms21030835 (PMC7038140; doi:10.3390/ijms21030835)
Supplement: Supplementary file 1 [file ijms-21-00835-s001.zip › Supplemental Table S1.docx]

**Supplemental Table 1.** Mutations in the *RPGR* gene found in the patients included in this study

| **Family-ID** | **cDNA change** | **Protein effect** | **Exon** | **Diagnosis** | **Reference** |
| --- | --- | --- | --- | --- | --- |
| A-1 | c.27del | p.(Asp10Ilefs*58) | 1 | RP | Talib et al. 2018 |
| B-2 | c.425T>G | p.(Ile142Ser) | 5 | RP | Talib et al. 2018 |
| B-3 | c.425T>G | p.(Ile142Ser) | 5 | RP | Talib et al. 2018 |
| C-4 | c.706C>T | p.(Gln236*) | 7 | RP | Buraczynska et al. 1997 |
| D-5 | c.2236_2237del | p.(Glu746Argfs*23) | ORF15 | RP | Vervoort et al. 2000 |
| E-6 | c.2323_2324del | p.(Arg775Glufs*59) | ORF15 | RP | Breuer et al. 2002 |
| F-7 | c.2838_2839del | p.(Glu947Glyfs*131) | ORF15 | RP | Pelletier et al. 2007 |
| G-8 | c.2840dup | p.(Glu949Glyfs*130) | ORF15 | RP | Neidhardt et al. 2008 |
| H-9 | c.3212-3218del | p.(Glu1071Alafs*16) | ORF15 | CRD | This study |
| Donor | c.3092del | p.(Glu1031Glyfs*58) | ORF15 | CRD | Demirci et al. 2002 |

Nucleotide changes in cDNA are noted with transcript NM_001034853.1 as reference. RP = retinitis pigmentosa; CRD = cone-rod dystrophy.
