# Supplementary material for: RPGR-Associated Dystrophies: Clinical, Genetic, and Histopathological Features
Source: Int J Mol Sci. 2020 Jan 28;21(3):835. doi: 10.3390/ijms21030835 (PMC7038140; doi:10.3390/ijms21030835)
Supplement: Supplementary file 1 [file ijms-21-00835-s001.zip › Supplemental Table S2.docx]

Supplemental Table 2. Retinal sensitivity values in patients with *RPGR*-associated dystrophies

| **Family-ID** | **Microperimetry** | | **Full-stimulus threshold** | | |
| --- | --- | --- | --- | --- | --- |
|  | **Mean Retinal sensitivity (dB)** | **Fixation stability OD/OS** | **Mean white (dB)** | **Mean blue-red difference** | **Mediated response** |
| A-1 | 3.20 | Unstable/Unstable | NP | NP | - |
| B-2 | 15.45 | Stable/Stable | NP | NP | - |
| B-3 | 24.35 | Stable/Stable | -43.77 | 21.86 | Mixed |
| C-4 | 5.45 | RU/RU | -10.72 | -1.00 | Cone |
| D-5 | 14.55 | Stable/Stable | -45.57 | 20.62 | Mixed |
| E-6 | 8.00 | Stable/NP | -16.87 | 6.31 | Mixed |
| F-7 | 11.50 | Stable/Stable | NP | NP | - |
| G-8 | 3.10 | NP/Stable | -8.89 | -.71 | Cone |
| H-9 | 20.50 | Stable/Stable) | -60.98 | 20.23 | Mixed |

RU = relatively unstable fixation; NP = not performed. Due to technical reasons, not all tests were performed in all patients. The difference in thresholds between blue and red stimuli determined whether responses were either: rod-mediated (difference of > 22dB), cone-mediated (difference of < 3dB) or mixed (between 3- 22 dB).
